# Maternal serum anti-Müllerian hormone in Sudanese women with preeclampsia

**DOI:** 10.1186/s13104-017-2544-6

**Published:** 2017-06-24

**Authors:** Eiman Agabain, Hameed Mohamed, Anas E. Elsheikh, Hamdan Z. Hamdan, Ishag Adam

**Affiliations:** 1 Medical College, Qassim University, Buraydah, Kingdom of Saudi Arabia; 2 Faculty of Medicine, Alneelain University, Khartoum, Sudan; 3 Omdurman University, Omdurman, Sudan; 4 Faculty of Medicine, University of Khartoum, P.O. Box 102, 11111 Khartoum, Sudan

**Keywords:** Anti-Müllerian hormone, Preeclampsia, Pregnancy, Predictors, Sudan

## Abstract

**Objectives:**

A case–control study was conducted at Omdurman Maternity Tertiary Hospital, Sudan, during the period from May to August 2014 to investigate AMH level in women with preeclampsia compared to healthy controls. The cases were women with preeclampsia and healthy pregnant women were the controls. The obstetrics and medical history was gathered using a questionnaire. AMH level was measured using ELISA.

**Results:**

There was no significant difference between the two groups (40 in each arm of the study) in the age, parity and gestational age. Thirty-three of the 40 cases were patients with severe preeclampsia. There was no significant difference in median inter-quartile of the AMH level between the women with preeclampsia and the controls [0.700 (0.225–1.500) vs. 0.700 (0.400–1.275) ng/ml, P = 0.967]. In a linear regression model there was no association between the log of AMH and age, parity, gestational age, BMI, hemoglobin level and preeclampsia.

## Introduction

Preeclampsia is a worldwide major health problem characterized by the occurrence of hypertension and proteinuria after 20 weeks of pregnancy in previously normotensive women [1]. Preeclampsia is a main factor for maternal and perinatal morbidity and mortality, where it is responsible for at least 9% of the maternal mortality that occur in Africa [2, 3]. Moreover various long-term effects on patients’ health, e.g. cardiovascular risk factors and premature vascular aging that could modify the ovarian aging process have been reported with preeclampsia [4, 5].

Anti-Müllerian hormone (AMH) is a heavily glycosylated glycoprotein produced by the gonads and it is involve in the development and differentiation of the reproductive system [6, 7]. AMH is a reliable predictor of ovarian reserve, in assisted reproduction outcomes such as stillbirth, preeclampsia, gestational diabetes mellitus, delivery of small for gestational age [8–11]. The AMH levels have recently been investigated in preeclampsia with inconsistent results, where some reports showed high levels and others showed low levels of AMH among women with preeclampsia [12–14]. There is a need to investigate whether AMH level during pregnancy can be used as a predictor for preeclampsia and its poor maternal/perinatal outcomes. Preeclampsia/eclampsia is the leading cause of obstetric complications and maternal mortality in Sudan [15–18]. The current study was conducted at Omdurman Maternity Hospital, Sudan to determine the level of AMH in women with preeclampsia and to add on our recent research on preeclampsia in Sudan [19–21].

## Main text

A case–control study was conducted at Omdurman Maternity Tertiary Hospital, Sudan during the period from May to August 2014. The cases were pregnant women diagnosed with preeclampsia. Preeclampsia was defined as the occurrence of hypertension (systolic blood pressure ≥140 mmHg or diastolic blood pressure ≥90 mmHg) after 20 weeks of gestation in a woman who is normotensive before, and proteinuria (presence of 300 mg or more of protein in 24 hours urine sample or ≥2+ on dipstick) [22]. The cases of preeclampsia were considered mild or severe according to the diastolic blood pressure of <110, or ≥110 mmHg respectively. The controls were healthy pregnant women that matched with the cases for gestational age. Women with thyroid disease, hypertension, renal disease, diabetes, liver disease and those who received medication for hypertension were excluded from the study.

After signing an informed consent, the details of obstetrics history (age, parity, and gestational age) was gathered and recorded. Weight and height were measured and body mass index (BMI) was calculated as weight in kilograms divided by the square of height in meters. Systolic and diastolic blood pressures were assessed in supine position using mercury sphygmomanometer by the same investigator.

Five milliliters of venous blood were taken from each woman (case or control) in a plain tube, allowed to clot, centrifuged, and stored at −20 °C (for 6 months) until analyzed by Ultra-Sensitive AMH/MIS ELISA KIT, (bioactiva diagnostica GmbH—Bad Homburg, Germany), where the manufacturers` instructions were followed. The samples were withdrawn from the cases at presentation which was at mean (SD) of 36.9 (1.0) weeks. The samples were run in duplicate and the mean of the two readings was taken. The reference interval for the KIT was 0.13–13.01 ng/ml.

A total sample of 40 participants in each arm of the study was calculated to investigate the mean difference of the AMH. This sample size would provide 80% power to detect a 5% difference at α = 0.05, with an assumption that complete data might not be available for 10% of participants.

SPSS for Windows (version 16.0) was used for data analyses. Continuous variables were checked for normality and their difference was compared between two groups using *T* test and Mann–Whitney U when the data were normally and not normally distributed, respectively. Linear regression analyses were performed where the log of AMH (not normally distributed) was the dependent variable, and age, parity, gestational age, maternal BMI and hemoglobin were the independent variables. P < 0.05 was considered statistically significant.

**Fig. 1:**
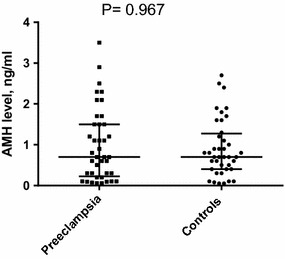
AMH level in women with preeclampsia and controls

The two groups (40 in each arm of the study) were matched in their basic characteristics, where there was no significant difference in the mean (SD) of the age, parity and gestational age [36.7 (1.9) vs. 36.9 (1.0) weeks, P = 0.625] between the studied groups. Thirty-three of the 40 cases were suffering from severe preeclampsia. All patients had late on-set preeclampsia (after 34 weeks of gestation). The BMI was marginally higher, while hemoglobin level was significantly lower in women with preeclampsia compared to the controls, Table 1. There was no significant difference in median inter-quartile of the AMH between the women with preeclampsia and the healthy controls [0.700 (0.225–1.500) vs. 0.700 (0.400–1.275) ng/ml, P = 0.967], Fig. 1.

**Table 1:** Mean (SD) of the basic characteristics in the cases and controls

Variables	Cases (N = 40)	Controls (N = 40)	P
Age, years	27.7 (6.9)	29.5 (6.7)	0.254
Parity	2.2 (2.3)	3.2 (2.8)	0.089
Gestational age, weeks	36.7 (1.9)	36.9 (1.0)	0.625
Body mass index, kg/m^2^	30.0 (5.8)	27.2 (4.9)	0.066
Hemoglobin, g/dl	10.9 (0.9)	11.7 (1.3)	0.004

In a linear regression model there was no association between the log of AMH and age, parity, gestational age, BMI, hemoglobin level and preeclampsia, Table 2.

**Table 2:** Linear regression analysis of factors associated with log of anti-Müllerian hormone AMH

Variable	Log of anti-Müllerian hormone		
	Coefficient	SE	P
Age, years	−0.022	0.015	0.933
Parity	0.020	0.035	0.158
Gestational age, weeks	−0.002	0.055	0.567
Body mass index, kg/m^2^	−0.005	0.015	0.977
Hemoglobin, g/dl	0.072	0.064	0.722

The main two findings of the current study were that first there was no significant difference in the AMH level between women with preeclampsia and the healthy controls. Secondly, significant association between AMH level and age, parity, gestational age and BMI was not established. This is similar with the findings of the recent studies that proved no significant difference in the AMH level between women with a history of preeclampsia and their matched control group [23, 24]. Furthermore, Birdir et al. [12] reported that at early gestation (11–13 weeks), the maternal serum (multiple of the expected median) values of AMH were not significantly different in pregnant women who developed preeclampsia and those who remained normotensive throughout pregnancy. They concluded that AMH was not an effective early predictor for the development of preeclampsia. Interestingly, in the later report the uncorrected median serum concentrations of AMH were significantly higher in the women with preeclampsia than in the controls [12].

Tokmak et al. [14], reported that AMH level (at delivery) was lower in preeclamptic patients than in normal pregnant women and there was no relationship between AMH level and adverse maternal (eclampsia, persistent hypertension and hemolysis, elevated liver enzyme and low platelets, and perinatal outcomes (prematurity, hypoglycemia, polycythemia, respiratory distress syndrome and perinatal deaths). It has been demonstrated that women with a history of preeclampsia had significantly lower AMH levels than women with normotensive pregnancies [5]. Remarkably, women with an AMH (in the first trimester) less than the 10th centile were at 3.3-fold increased risk of pregnancy-induced hypertension. However, there were no significant associations between low AMH concentration and adverse maternal or perinatal outcomes [13].

In the current study, AMH level was not associated with age, parity, gestational age and BMI, which is comparable with the findings of a recent report [12].

It is worth to be mentioned that, to our knowledge, this is the first published data on AMH serum level and preeclampsia in Africa. Maternal serum concentration of AMH was reported to be increased in Afro-Caribbean women [12], while African women (probably due to genetic or environmental factors) showed a relatively high ovarian reserve, AMH and oocyte yield compared to other races [25]. AMH has advantages over other ovarian reserve marker; it has no significant intra/inter-cycle variability, therefore it can be measured on any day of the cycle [26].

Thus the current study showed no significant difference in AMH level between women with preeclampsia and the controls. AMH level was not associated with age, parity, gestational and BMI.

## Limitations

One of the limitations of the current study is that the patients were not followed-up to the postpartum period. The follow-up would have detected the post-partum changes of the AMH level and would have compared our results with the previous studies that were conducted following delivery. All women had late onset preeclampsia and none of them had early preeclampsia and this is the second limitation of the current study. Furthermore neither Doppler ultrasound nor intrauterine growth restriction were looked for.

## Abbreviations

AMH: anti-Müllerian hormone
BMI: body mass index
